# Artificial intelligence in orthodontics: clinical maturity, evidence gaps, and emerging applications—a narrative review

**DOI:** 10.1007/s44445-026-00249-6

**Published:** 2026-10-09

**Authors:** Kanza Chandio, Laiba Iftikhar, Anum Shehzad, Ifrah Rao, Minahil Waseem, Muhammad Anas, Muhammad Farrukh, Ammar Al Shalabi, Sameh Talaat, Sinan Sen, Fatma Hassanein, Tarek Elshazly

**Affiliations:** 1 Ziauddin University, Karachi, Pakistan; 2 Dow International Dental College, Karachi, Pakistan; 3 Shaheed Zulfikar Ali Bhutto Institute of Science and Technology University, Karachi, Pakistan; 4 Rashid Latif Dental College, Lahore, Pakistan; 5 Army Medical College, Rawalpindi, Pakistan; 6 Bacha Khan Dental College, Mardan, Pakistan; 7 Khyber Medical University, Peshawar, Pakistan; 8 Margalla Institute of Health Sciences, Rawalpindi, Pakistan; 9 Hamdan Bin Mohammed College of Dental Medicine (HBMCDM), Mohammed Bin Rashid University of Medicine and Health Sciences (MBRU), Dubai, United Arab Emirates; 10 Future University in Egypt, Cairo, Egypt; 11 University Hospital Schleswig-Holstein, Kiel, Germany; 12 King Salman International University, El-Tor, Egypt; 13 University Hospital Bonn, Bonn, Germany

**Keywords:** Orthodontics, Artificial intelligence, Machine learning, Computer-assisted diagnosis, Telemedicine

## Abstract

Artificial intelligence (AI) is rapidly transforming orthodontics by enhancing diagnostic accuracy, supporting clinical decision-making, and facilitating digital workflows. This narrative review provides a comprehensive overview of current AI technologies and their applications in orthodontics, including diagnostic imaging, treatment planning, treatment monitoring, patient communication, education, and emerging generative AI tools. The available evidence indicates that automated cephalometric landmark detection and cone-beam computed tomography (CBCT) segmentation have among the most advanced technical and external-validation evidence bases in orthodontic AI, whereas digital-model analysis has demonstrated high technical performance but more limited independent validation. However, no application met the predefined criteria for higher clinical maturity because prospective clinical effectiveness remained insufficiently established. Other emerging or experimental applications include AI-assisted treatment planning, remote monitoring, large language models (LLMs), and robotic systems, for which evidence remains predominantly retrospective, technical, feasibility-based, or early clinical. However, much of the current evidence is derived from retrospective studies, technical validation investigations, and proof-of-concept reports, with limited prospective clinical data demonstrating improvements in treatment quality, efficiency, or long-term patient outcomes. Important challenges remain regarding data quality, algorithmic bias, external validation, explainability, patient privacy, and regulatory oversight. Overall, AI should currently be regarded as a clinician-supervised decision-support tool rather than a replacement for professional expertise. Future progress is likely to depend on robust prospective clinical trials, transparent reporting, and responsible integration of AI into evidence-based orthodontic practice and the broader digital transformation of dentistry.

## Highlights

AI is transforming orthodontic diagnosis, treatment planning, and digital workflows.

Diagnostic imaging has the most advanced technical and external-validation evidence among orthodontic AI applications.

AI-assisted monitoring remains emerging, while several LLM and robotic applications remain experimental.

Current evidence is dominated by technical validation and retrospective studies.

Prospective clinical effectiveness and meaningful clinical benefit remain major evidence gaps.

## Introduction

**Fig. 1:**
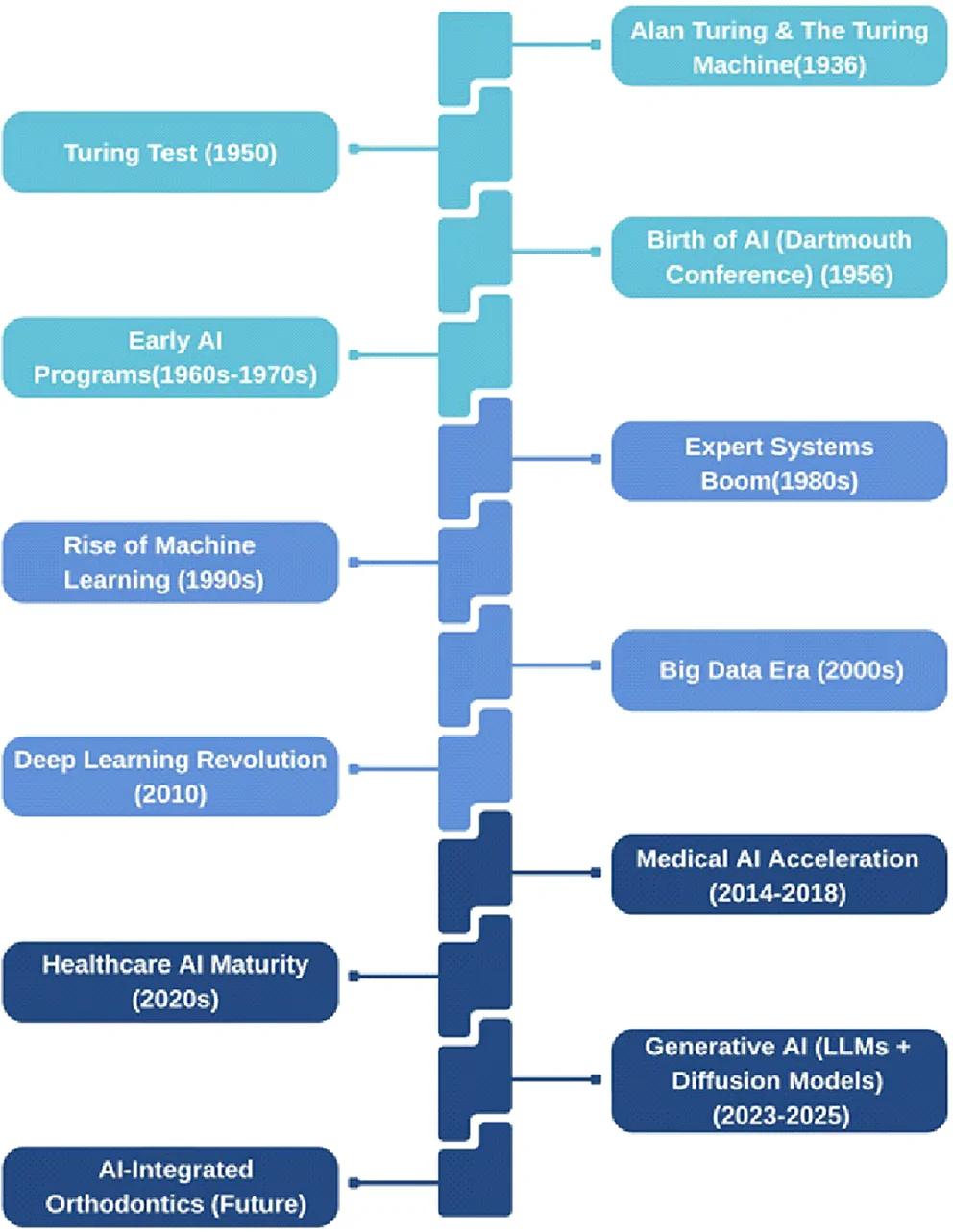
Evolution of orthodontic technology from conventional appliances to contemporary digital workflows and potential future AI-integrated systems. The final stage represents a conceptual projection of possible future integration rather than an established or validated trajectory toward routine clinical implementation. Figure created by the authors

Artificial intelligence (AI) refers to computer-based systems capable of learning, reasoning, and decision-making through advanced digital technologies (Adel et al. 2021, Akdeniz and Tosun 2021, Hung et al. 2020). While the foundational principles of simulating human cognition trace back to Alan Turing (Turing 1950), early AI approaches were largely rule-based, relying on predefined rules and algorithms (Samaranayake et al. 2025). In contrast, modern AI is increasingly data-driven, analyzing large datasets to identify patterns and optimize performance, enabling applications such as natural language processing (NLP) and modeling and automated image analysis (Yu et al. 2023) (Fig. 1).

As a core subset of AI, machine learning (ML) excels at pattern recognition within complex datasets (Bichu et al. 2021). Driven by increased computational power and the evolution of artificial neural networks (ANNs), including convolutional neural networks (CNNs) for image analysis and generative adversarial networks (GANs) for synthetic data generation, deep learning (DL) has substantially improved performance on narrowly defined image-analysis tasks, in some cases approaching the agreement observed between human examiners. Reported performance, however, depends on the specific task, dataset, reference standard, performance metric, and validation setting and should not be generalized across clinical application (Albalawi and Abalkhail 2022, Litjens et al. 2017). AI may also be integrated with complementary digital technologies, including virtual reality (VR), augmented reality (AR), and Internet of Things (IoT)–enabled devices, to support applications such as treatment visualization and remote monitoring (Uruthiralingam and Rea 2020, Merenda et al. 2020, Kapila et al. 2023) (Table 1).

**Table 1:** Classification and functional definitions of artificial intelligence approaches and complementary digital technologies relevant to orthodontics

Category	AI Technology	Definition and Dental Application
Core AI & Learning	**Artificial Intelligence (AI)**	Broad field of computer systems designed to perform tasks requiring human intelligence, including diagnosis, decision-making, and pattern recognition.
	**Machine Learning (ML)**	Subset of AI in which algorithms learn from data to improve predictions or decisions, such as treatment outcome prediction and growth assessment.
	**Deep Learning (DL)**	Specialized form of ML using multi-layered neural networks to process complex patterns.
Neural Architectures	**Artificial Neural Network (ANN)**	Foundational architecture inspired by biological neural networks; consists of interconnected nodes used for classification and prediction.
	**Convolutional Neural Network (CNN)**	Deep learning architecture designed for image recognition and analysis, including tooth segmentation in CBCT and radiographs.
	**Generative Adversarial Network (GAN)**	Deep learning model in which two networks compete to generate realistic synthetic data, such as simulated post-treatment facial outcomes.
Sensory & Language	**Computer Vision (CV)**	AI field enabling computers to interpret visual data, including radiographs, intraoral photographs, and scans.
	**Natural Language Processing (NLP)**	AI techniques enabling machines to understand and analyze human language, with applications in clinical documentation and patient communication.
Complementary Digital Technologies	**Augmented Reality (AR)**	Technology that overlays digital information onto the real clinical environment, such as virtual bracket positioning.
	**Virtual Reality (VR)**	Fully immersive, computer-generated environment used for virtual surgical simulation and 3D treatment planning.
	**Internet of Things (IoT)**	Network of interconnected devices that exchange data, which may support sensor-based or remote orthodontic monitoring when integrated with appropriate sensing and analytical systems.

Note. AR, VR, and IoT are complementary digital technologies that may be integrated with AI-enabled orthodontic systems but are not themselves AI methods. Abbreviations: AI, artificial intelligence; ML, machine learning; DL, deep learning; ANN, artificial neural network; CNN, convolutional neural network; GAN, generative adversarial network; CV, computer vision; NLP, natural language processing; AR, augmented reality; VR, virtual reality; IoT, Internet of Things; CBCT, cone-beam computed tomography

**Fig. 2:**
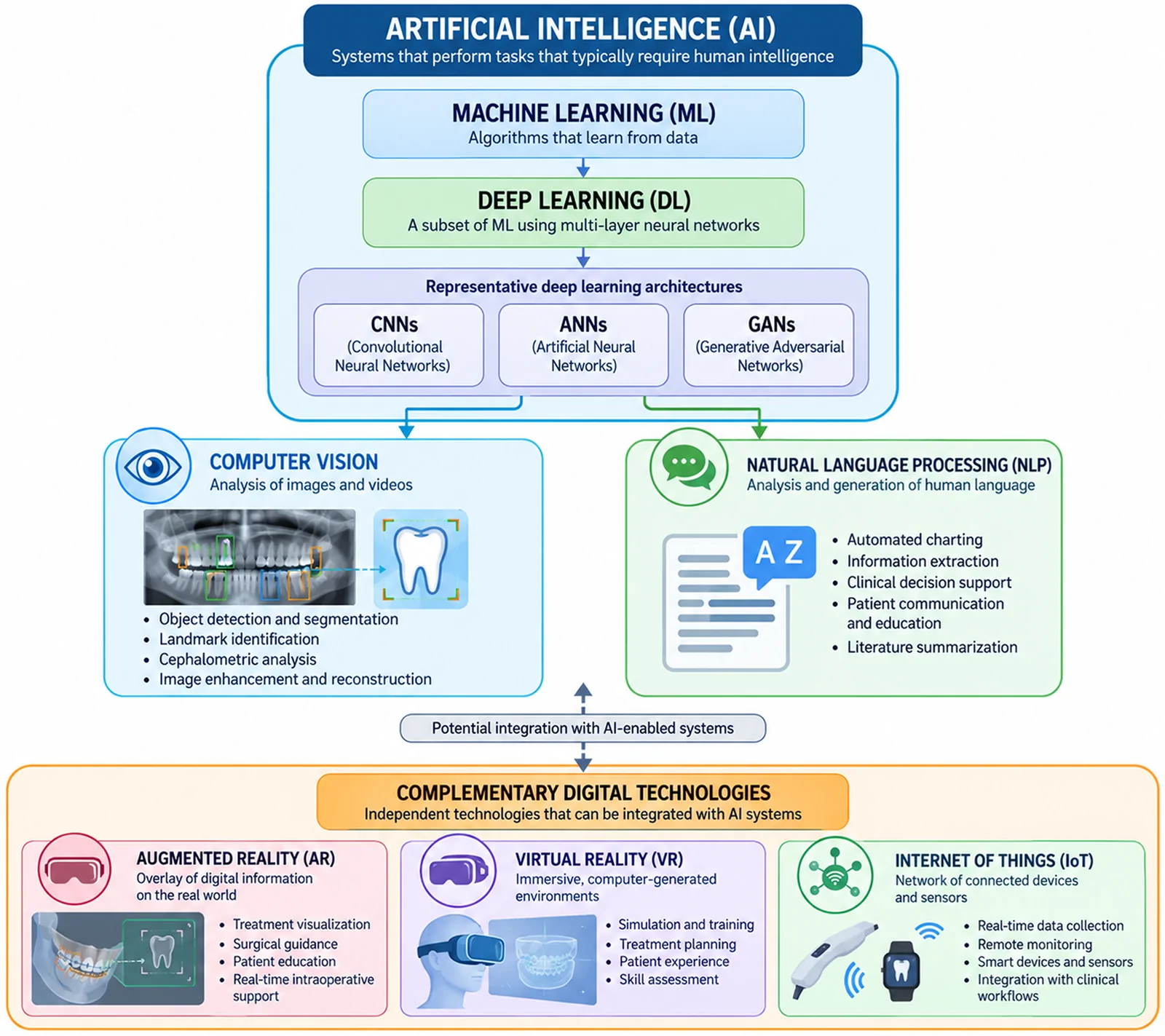
Artificial intelligence approaches and complementary digital technologies relevant to orthodontics. Artificial intelligence encompasses machine learning (ML) and deep learning (DL), with representative neural network architectures including artificial neural networks (ANNs), convolutional neural networks (CNNs), and generative adversarial networks (GANs), and applications including computer vision and natural language processing. Augmented reality (AR), virtual reality (VR), and the Internet of Things (IoT) are shown as complementary digital technologies that may integrate with AI-enabled systems but are not themselves subclasses of artificial intelligence. Figure created by the authors

In modern healthcare, AI has been applied to diagnostic image analysis and predictive modeling, including the identification of patterns in medical images (Albalawi and Abalkhail 2022, Liu et al. 2023, Dipalma et al. 2023). Beyond diagnostics, AI-enabled robotics has also been investigated for workflow automation and assistance in selected surgical and procedural tasks (Adel et al. 2021). These developments have expanded the potential applications of AI in dentistry and orthodontics (Akdeniz and Tosun 2021, Hung et al. 2020, Bichu et al. 2021, Albalawi and Abalkhail 2022). Orthodontic AI applications have expanded from diagnostic imaging to malocclusion analysis, facial symmetry assessment, and decision support related to extractions, orthognathic surgery, and clear aligners; although performance varies substantially across these tasks (Fig. 4) (Bichu et al. 2021, Gracea et al. 2025). Functioning as a decision-support tool rather than a replacement for clinicians, AI-driven cephalometric systems have demonstrated strong agreement with conventional tracing for selected cephalometric measurements, although performance varies across parameters and platforms, datasets, and validation settings (Bor et al. 2024, Kazimierczak et al. 2024) (Fig. 2). However, widespread clinical adoption requires addressing critical ethical and governance challenges. Robust safeguards are needed to protect patient data, ensure appropriate data governance, and mitigate algorithmic bias, while effective oversight is essential to promote equitable and trustworthy AI implementation (Obermeyer et al. 2019, Price and Cohen 2019).

Given the rapid development and increasing investigation of AI across orthodontic workflows, a structured review of current evidence is essential to understand its clinical value, identify knowledge gaps, and guide future research. Alongside these clinical developments, a growing set of reporting and evaluation frameworks has emerged to improve the transparency, methodological rigor, clinical evaluation, and trustworthiness of healthcare AI research, including Transparent Reporting of a multivariable prediction model for Individual Prognosis Or Diagnosis + Artificial Intelligence (TRIPOD + AI) (Collins et al. 2024) for clinical prediction models, Standards for Reporting of Diagnostic Accuracy Studies - Artificial Intelligence (STARD-AI) (Sounderajah et al. 2025) for AI-based diagnostic accuracy studies, Developmental and Exploratory Clinical Investigations of DEcision support systems driven by Artificial Intelligence (DECIDE-AI) (Vasey et al. 2022) for early-stage clinical evaluation of AI-driven clinical decision-support systems, FUTURE-AI (Lekadir et al. 2025) for trustworthy and deployable healthcare AI, and, for generative AI and large language model applications, Transparent Reporting of a multivariable prediction model for Individual Prognosis Or Diagnosis - Large Language Models (TRIPOD-LLM) (Gallifant et al. 2025) and Chatbot Assessment Reporting Tool (CHART) (The CHART Collaborative 2025). These frameworks provide contemporary reference points for interpreting reporting, validation, and translational gaps in orthodontic AI research; however, formal assessment of study-level adherence to these frameworks was beyond the scope of this narrative review.

Previous scoping and systematic reviews have mapped AI applications in orthodontic diagnosis and treatment planning (Bichu et al. 2021, Gracea et al. 2025, Monill-Gonzalez et al. 2021) and in clear aligner therapy (Ruiz et al. 2025). Although these reviews have characterized the available technologies and their performance across specific orthodontic applications, an important translational question remains: technical performance alone does not establish external generalizability, prospective clinical effectiveness, or clinically meaningful benefit. Accordingly, there remains a need to synthesize orthodontic AI evidence according to its stage of clinical maturity and to interpret the evidence in the context of contemporary AI reporting and evaluation frameworks. The present review differs by adopting this clinical-translation perspective: applications are classified by clinical maturity using predefined evidence criteria (Sect.  2.5), while contemporary reporting and evaluation frameworks provide context for interpreting methodological and reporting gaps relevant to clinical translation.

Accordingly, the objectives of this narrative review are:

1. To provide a structured overview of AI-based technologies applied in orthodontics, including ML, DL, computer vision (CV), and NLP.
2. To critically appraise the clinical maturity of major AI applications across orthodontic workflows by distinguishing technical performance, external validation, prospective clinical effectiveness, and demonstrated clinical or patient benefit.
3. To identify the principal evidence, reporting, methodological, ethical, legal, and implementation gaps that currently limit the clinical translation of AI in orthodontics.
4. To examine emerging AI applications and their integration with emerging digital technologies and identify priorities for clinically meaningful and responsibly implemented AI research in orthodontics.

## Materials and methods

### Study design

This study was conducted as a narrative literature review supported by a structured and transparent literature search. Although it was not designed or registered as a systematic review, established methodological principles were applied to the search, selection, and reporting stages in order to improve transparency and reproducibility. No protocol was registered.

### Search strategy

An electronic literature search was performed in PubMed, ScienceDirect, and the Cochrane Library. PubMed was selected as the principal bibliographic database for biomedical and dental literature, and the Cochrane Library was searched to identify controlled trials and systematic reviews. ScienceDirect was searched as a publisher platform rather than as a bibliographic database, to capture peer-reviewed engineering and computational literature relevant to orthodontic applications that is less consistently indexed in biomedical databases. The absence of Scopus, Embase, and Web of Science may have limited literature coverage and is acknowledged in the Limitations section. The search covered publications from 1 January 2000 to 31 August 2026, reflecting the period during which ML and DL applications underwent substantial development. The final search was executed on 31 August 2026. Full database-specific search strings, including fields and filters, are presented in Table 2. To supplement the database search, the reference lists of all included articles and of relevant reviews were screened manually for additional publications.

### Eligibility criteria

Publications were eligible for inclusion if they met all of the following criteria:

- described a clearly defined AI application, including ML, DL, CV, NLP, or generative models;
- addressed a task with direct relevance to orthodontic diagnosis, treatment planning, treatment delivery, monitoring, patient communication, education, or workflow;
- reported original research, clinical or technical validation, systematic or scoping review;
- were published in English between 1 January 2000 and 31 August 2026.

Publications were excluded if they:

- did not describe an identifiable AI application;
- addressed dentistry or medicine without a demonstrable orthodontic application;
- were editorials, commentaries, letters, or conference abstracts without accompanying full-text data;
- were preprints, dissertations, theses, non-peer-reviewed technical reports, or other non-peer-reviewed sources.

Non-peer-reviewed sources, including preprints, dissertations, and theses, were excluded from the evidence synthesis. Peer-reviewed conference proceedings and edited book chapters were retained where they constituted the original publication of a described system or dataset, as is conventional in computer science. Methodological reporting guidelines, regulatory documents, and other authoritative background sources were used only to contextualize reporting, governance, and regulatory issues and were not considered part of the orthodontic AI evidence synthesis.

### Study selection and data extraction

Records retrieved from all sources were exported to Zotero and de-duplicated. Titles and abstracts were screened by two reviewers against the eligibility criteria, with disagreements resolved by discussion and a third author consulted where consensus could not be reached. Potentially eligible records underwent full-text assessment against the same eligibility criteria. Retracted publications identified through the Retraction Watch integration in Zotero were excluded.

For publications contributing to the evidence synthesis, data were extracted on the AI application, clinical task, study design, study setting and population or dataset, AI model or system where reported, comparator or reference standard, principal performance or clinical outcomes, validation setting, and evidence of prospective clinical evaluation or clinically meaningful benefit. For generative AI and LLM studies, model identity and version and the timing of evaluation were additionally recorded where reported. These data informed the application-specific narrative synthesis and clinical-maturity appraisal described in Sect.  2.5 and 2.6. Data extraction was performed by two reviewers, with disagreements resolved through discussion and, where necessary, consultation with a third author.

The search retrieved 2,022 records (PubMed, *n* = 1,699; ScienceDirect, *n* = 204; Cochrane Library, *n* = 119). After removal of 169 duplicates, 1,853 records were screened, of which 1,808 were excluded during title/abstract screening or subsequent full-text assessment because they did not meet the predefined eligibility criteria. Forty-five publications identified through the database search were included in the evidence synthesis.

**Fig. 3:**
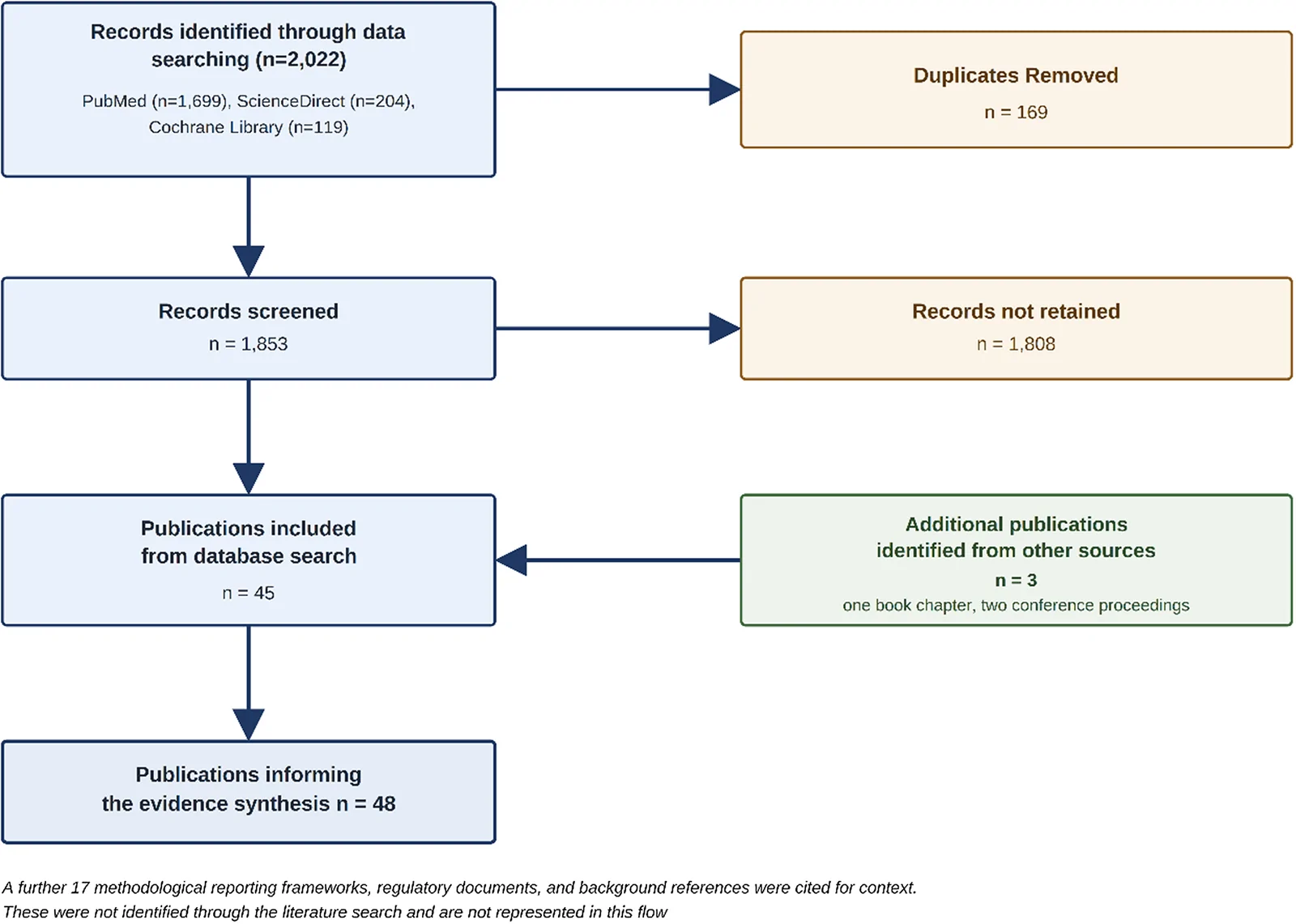
Flow of literature identification and selection. Records were identified through searches of PubMed, ScienceDirect, and the Cochrane Library, de-duplicated in Zotero, and screened against the eligibility criteria described in Sect.  2.3. The diagram was adapted from the PRISMA 2020 flow structure to transparently report literature identification and selection; its use does not indicate that the present narrative review was conducted as a systematic review. Figure created by the authors

A further 3 publications, comprising one book chapter and two peer-reviewed conference proceedings not indexed in the databases searched, were identified through reference-list screening, giving a total of 48 publications informing the evidence synthesis. In addition, 17 methodological reporting frameworks, regulatory documents, and background references on AI, medical imaging, and research ethics were cited for context; these were not identified through the literature search and were not included in the selection flow (Fig. 3).

### Classification of clinical maturity

Applications were assigned to one of three clinical-maturity categories using predefined criteria applied independently by two authors, with disagreements resolved through discussion and, where consensus could not be reached, adjudication by a third author.

Four evidence levels were assessed for each application:

1. Technical performance: task-specific quantitative performance evaluated against an appropriate reference standard in development, test, or internal-validation datasets;
2. External validation: evaluation in data, populations, institutions, acquisition environments, or clinical settings distinct from those used for model development;
3. Prospective clinical effectiveness: prospective evaluation during clinical use, examining effects on clinical decision-making, diagnostic or treatment performance, workflow, safety, or other clinically relevant outcomes; and.
4. Demonstrated clinical or patient benefit: comparative evidence demonstrating improvement in clinically meaningful patient, clinician, workflow, or health-system outcomes rather than technical performance alone.

Applications were classified as having higher clinical maturity where technical performance was supported by external validation and at least some prospective clinical evaluation; as emerging where technical performance had been demonstrated and external validation was available or developing, but prospective clinical effectiveness and clinically meaningful benefit remained insufficiently established; and as experimental where evidence was restricted predominantly to proof-of-concept, feasibility, laboratory, simulation, or early technical-validation studies without convincing external or prospective clinical validation.

Commercial implementation was recorded separately as an implementation characteristic rather than as evidence of clinical effectiveness or superiority. Where the available evidence differed materially between distinct uses of the same technology, those uses were classified separately. The resulting classification, with supporting references and a brief evidence-based justification for each assignment, is presented in Table 3.

### Evidence synthesis

The identified literature was synthesized qualitatively, with emphasis on AI technologies, clinical applications, study design and evidence characteristics, validation setting, external validation, prospective clinical evaluation, reported clinical or patient benefit, translational maturity, current limitations, and future research directions. Owing to the heterogeneity of study designs, reference standards, and outcome measures, no quantitative synthesis or meta-analysis was performed.

Contemporary reporting and evaluation frameworks for healthcare AI research, including TRIPOD + AI, STARD-AI, DECIDE-AI, FUTURE-AI, and, for generative applications, TRIPOD-LLM and CHART (Collins et al. 2024, Sounderajah et al. 2025, Vasey et al. 2022, Lekadir et al. 2025, Gallifant et al. 2025, The CHART Collaborative 2025), were used as methodological reference points to contextualise reporting and translational gaps in the orthodontic literature. They were not applied as formal appraisal instruments, and no individual study was formally scored for adherence to their individual reporting items. No formal risk-of-bias or methodological-quality assessment was undertaken; this is acknowledged in the Limitations section. As this review was based exclusively on previously published literature, ethical approval was not required.

**Table 2:** Database-specific literature search strategies, fields, filters, and records retrieved

Database (interface)	Search string as executed, with fields and filters	Records retrieved
PubMed (NCBI)	(“Artificial Intelligence“[MeSH] OR “artificial intelligence“[tiab] OR “machine learning“[tiab] OR “deep learning“[tiab] OR “computer vision“[tiab] OR “natural language processing“[tiab] OR “generative AI“[tiab] OR “large language model*“[tiab] OR “ChatGPT“[tiab]) AND (“Orthodontics“[MeSH] OR orthodontic*[tiab] OR cephalometr*[tiab] OR “clear aligner*“[tiab]) *Filters: publication date 1 January 2000 to 31 August 2026. No language or article-type filter applied.*	1,699
ScienceDirect (Elsevier)	**Search 1**: (“artificial intelligence” OR “machine learning” OR “deep learning”) AND (orthodontics OR cephalometry) **Search 2**: (“computer vision” OR “natural language processing” OR “neural network”) AND (orthodontics OR cephalometry) **Search 3**: (“generative AI” OR “large language model” OR “ChatGPT”) AND (orthodontics OR “clear aligner”) *Fields: Title*,* abstract*,* or author-specified keywords. Filters: years 2000–2026; article types Research articles and Review articles. Searches were split because the ScienceDirect advanced search interface permits a maximum of eight Boolean connectors per query and does not support wildcard truncation.*	204
Cochrane Library	(“artificial intelligence” OR “machine learning” OR “deep learning” OR “neural network*” OR “ChatGPT” OR “large language model*”):ti, ab, kw AND (orthodontic* OR cephalometr* OR “clear aligner*”):ti, ab, kw *Filters: Cochrane Reviews and Trials; publication years 2000–2026.*	119
**Total retrieved**		**2**,**022**

Note. The final search was conducted on 31 August 2026. Abbreviations: MeSH, Medical Subject Headings; NCBI, National Center for Biotechnology Information; ti, ab, kw, title, abstract, keywords fields; tiab, title/abstract field tag

## Artificial intelligence technologies

AI encompasses a range of computational approaches that enable computers to analyze data, recognize patterns, and generate predictions, classifications, or other outputs that may support clinical tasks. In orthodontics, the most relevant technologies include ML, DL, NLP, CV, and, more recently, generative AI and large language models (LLMs). Together, these technologies underpin a broad range of investigated applications, from automated image analysis and prediction to clinical documentation and patient communication.

### Machine Learning (ML)

Within AI systems, ML is a computational approach that enables algorithms to learn patterns from data without being explicitly programmed for every decision rule (Mohammad-Rahimi et al. 2021, Asiri et al. 2020). Based on their learning strategy, machine-learning methods are commonly categorized into supervised, unsupervised, and reinforcement learning (Mohammad-Rahimi et al. 2021, Asiri et al. 2020). In orthodontics, ML has been investigated for identifying patterns within clinical, imaging, and demographic datasets and for tasks related to diagnosis, treatment planning, and outcome prediction (Bichu et al. 2021, Dipalma et al. 2023, Mohammad-Rahimi et al. 2021). Model development typically involves data acquisition, feature extraction, training, and performance evaluation; importantly, internal validation assesses model performance within the development context, whereas external validation using independent data is required to evaluate generalizability beyond that context (Albalawi and Abalkhail 2022, Mohammad-Rahimi et al. 2021). However, ML performance is strongly influenced by the quality and representativeness of training data, and models developed from limited or biased datasets may generalize poorly to different clinical populations or acquisition settings (Fawaz et al. 2023, Nordblom et al. 2024). Accordingly, high performance during model development or internal validation should not, by itself, be interpreted as evidence of generalizable clinical effectiveness.

### Deep Learning (DL)

DL is a specialized subset of ML based on multilayer ANNs that automatically extract hierarchical features from input data, particularly medical images (Asiri et al. 2020, Büyük and Hatal 2019). CNNs, which are widely applied to orthodontic imaging tasks, have been investigated for image recognition, segmentation, and cephalometric landmark detection (Bichu et al. 2021, Schwendicke et al. 2021). Reported DL performance varies according to the task, dataset, reference standard, model architecture, and validation setting; therefore, superiority over conventional ML approaches or human examiners should not be generalized across applications (Gracea et al. 2025, Mohammad-Rahimi et al. 2021). Nevertheless, these models often require extensive, well-annotated training datasets and may have limited interpretability, particularly when the basis for individual predictions cannot be readily examined. These characteristics raise concerns regarding transparency, clinical accountability, dataset bias, and generalizability (Gracea et al. 2025, Fawaz et al. 2023, Nordblom et al. 2024). Moreover, strong technical performance in image classification, segmentation, or landmark detection does not necessarily demonstrate that use of the model improves clinical decisions, treatment outcomes, or patient benefit.

### Natural Language Processing (NLP)

NLP enables AI systems to interpret and analyze written or spoken language, and can be used to extract or structure information from unstructured clinical text (Yu et al. 2023). In orthodontics and healthcare more broadly, NLP has potential applications in automated documentation, electronic health record analysis, report generation, and extraction of clinically relevant information from patient records, although the extent to which these applications reduce workload or improve documentation quality requires application-specific clinical evaluation (Yu et al. 2023, Fawaz et al. 2023). NLP may also be applied to patient-generated text or feedback to identify information related to satisfaction, concerns, or treatment experience (Yu et al. 2023, Fawaz et al. 2023). However, variability in clinical terminology, documentation styles, language, and institutional practice remains a challenge, requiring appropriate validation before implementation in orthodontic workflows, with orthodontic-specific NLP evidence being currently minimal as most applications are extrapolated from general healthcare informatics (Yu et al. 2023, Fawaz et al. 2023, Salmanpour et al. 2025).

### Computer Vision (CV)

CV enables AI systems to analyze and interpret visual information from photographs, radiographs, videos, and three-dimensional (3D) scans. Most contemporary CV systems used for medical and dental image analysis employ DL architectures, particularly CNNs, for a range of image-analysis tasks, including image classification, object detection, and segmentation (Litjens et al. 2017, Yu et al. 2021). In orthodontics, CV has been investigated for automated facial analysis, cephalometric landmark identification, tooth segmentation, treatment monitoring, and digital smile simulation, with reported performance varying according to the specific task and validation setting (Bor et al. 2024, Kazimierczak et al. 2024, Ryu et al. 2022). Despite promising technical performance in selected applications, performance remains sensitive to image quality, acquisition protocols, dataset composition, annotation/reference standards, and population diversity, highlighting the need for standardized evaluation procedures and independent external validation (Nordblom et al. 2024, Bichu et al. 2025). Accordingly, technical accuracy in image recognition, segmentation, or landmark localization should be distinguished from evidence that CV improves clinician performance, treatment decisions, workflow, or patient outcomes.

### Generative AI and Large Language Models (LLMs)

Generative AI comprises models capable of generating new content, including text, images, and other data modalities, based on patterns learned during training. LLMs, including ChatGPT, Microsoft Copilot, and Gemini, have been evaluated in dentistry and orthodontics for patient communication, clinical documentation, education, scientific writing, and other information-processing tasks (Yu et al. 2023, Salmanpour et al. 2025, Dursun and Bilici Geçer 2024, Santonocito et al. 2025). Comparative evaluations to date have focused primarily on ChatGPT-4.0 and Microsoft Copilot (Salmanpour et al. 2025, Dursun and Bilici Geçer 2024), with one study additionally evaluating ChatGPT-3.5 and Gemini in orthodontic emergency scenarios (Erdem et al. 2025). Reporting of specific model releases and evaluation dates is inconsistent across these studies, limiting the reproducibility and temporal interpretability of the comparisons. However, performance reported for LLMs is inherently dependent on the specific model and version evaluated, the date of evaluation, prompt formulation, conversation context, and, for proprietary systems, provider-side updates. These characteristics may limit reproducibility and comparability between studies, particularly when model versions, evaluation dates, or prompting procedures are incompletely reported. In addition, performance on examination questions, clinical vignettes, or patient-information tasks should not be interpreted as evidence of clinical effectiveness without prospective evaluation in real-world clinical settings. Important limitations include factual inaccuracies and hallucinations, inconsistent responses, potential bias, privacy and confidentiality concerns, and uncertainty regarding accountability and regulatory oversight (Yu et al. 2023, Price and Cohen 2019). Evidence concerning LLMs requires cautious interpretation because performance is closely tied to the specific model evaluated. Proprietary models are updated frequently and without public versioning of their underlying parameters, so findings obtained with one release may not hold for its successor, and evaluations conducted only months apart may not be directly comparable. Outputs are additionally sensitive to prompt phrasing, conversational context, and sampling variability, such that identical questions may yield materially different responses on repeated administration. Because most commercial models cannot be accessed in a fixed, archivable state, independent replication of published evaluations is generally not possible. Studies evaluating LLMs should therefore report the exact model version, the interface used, the dates of evaluation, the prompts administered, whether repeated queries were performed, and the outcome-assessment method. Reporting guidelines such as TRIPOD-LLM and CHART have been proposed to standardize the reporting of such studies (Gallifant et al. 2025, The CHART Collaborative 2025). These constraints are particularly relevant where LLMs are used for direct patient counseling, since an inaccurate or outdated response may influence patient behavior before any clinician review occurs. Fluent and plausible outputs may contain inaccurate, incomplete, or contextually inappropriate information, and benchmark performance does not establish the safety of unsupervised patient counseling. Consequently, LLMs should be regarded as adjunctive technologies requiring appropriate clinician supervision rather than replacements for professional expertise (Salmanpour et al. 2025).

#### Current evidence and clinical translation

The clinical maturity of AI cannot be inferred from the technology class alone and varies substantially according to the specific orthodontic application. ML-, DL-, and CV-based systems have accumulated substantial technical-validation evidence for selected imaging tasks, whereas evidence of external generalizability, prospective clinical effectiveness, and patient benefit remains less consistent. NLP and generative AI applications are more recent, and their clinical evidence is currently more limited and particularly sensitive to system- and evaluation-specific factors. Across all technologies, future research should prioritize independent external validation and prospective multicenter clinical evaluation, standardized reporting, explainable AI, and evaluation of clinically meaningful patient, clinician, workflow, and health-system outcomes rather than relying predominantly on technical performance metrics. Accordingly, the clinical maturity of individual orthodontic AI applications is evaluated in the following sections using the predefined evidence criteria described in Sect.  2.5, distinguishing technical performance, external validation, prospective clinical effectiveness, and demonstrated clinical or patient benefit.

## Application of artificial intelligence technologies in orthodontics

### Applications with the most advanced clinical evidence

#### Diagnostic Imaging: cephalometric landmark detection, CBCT segmentation, and digital models

**Fig. 4:**
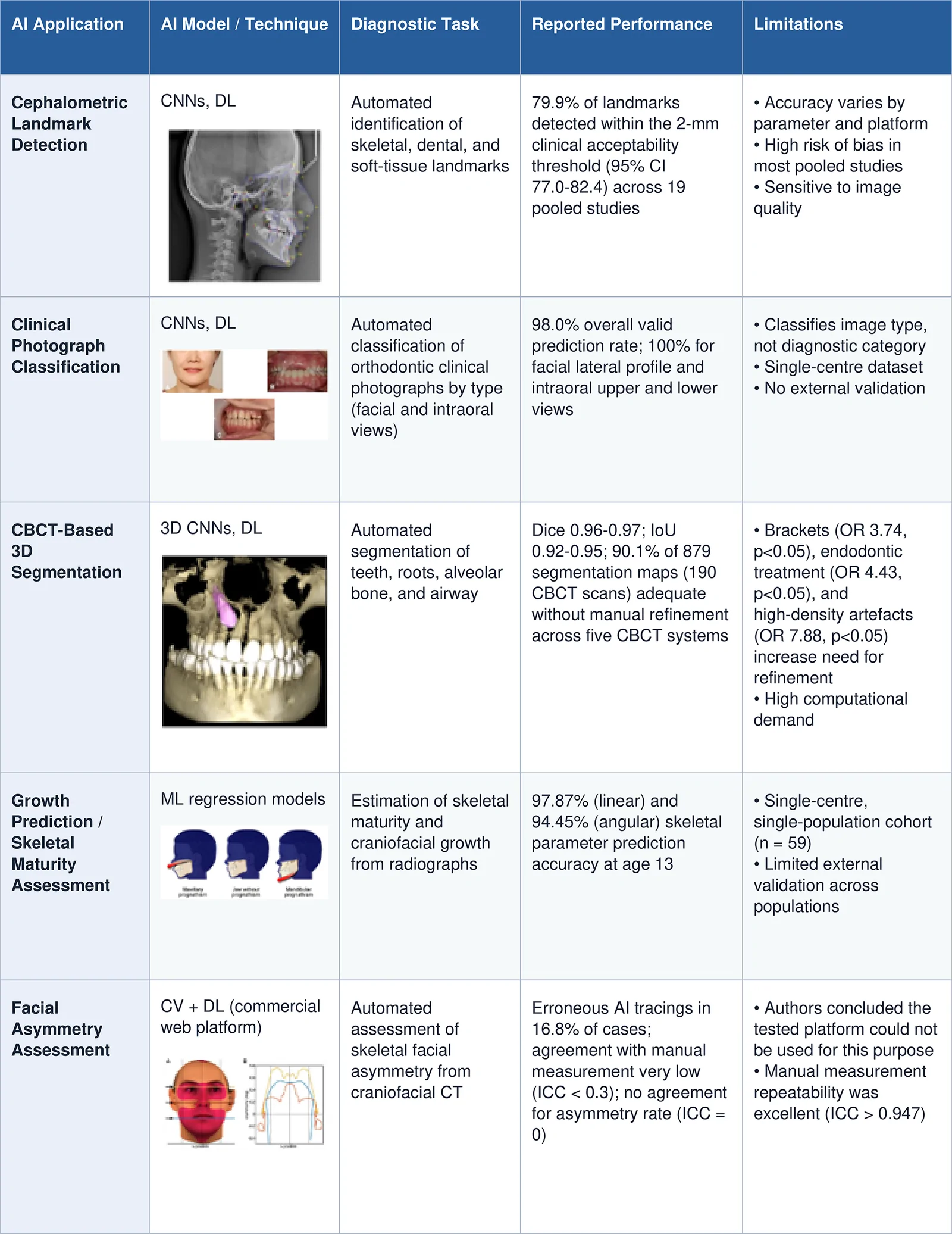
AI-based diagnostic applications in orthodontics: techniques, reported performance, and limitations. Performance data are drawn from the cited studies summarized in this section: cephalometric landmark detection from a systematic review and meta-analysis of 19 studies (Schwendicke et al. 2021); clinical photograph classification from deep-learning analysis of routine orthodontic photographs (Ryu et al. 2022); CBCT segmentation from a multicentre external validation study across five CBCT systems (Adiverci et al. 2026); skeletal maturity and craniofacial growth prediction from validation of machine-learning regression models (Kim et al. 2023, Kök et al. 2019); and facial asymmetry assessment from comparison of an AI-driven platform with manual 3D measurement (Kazimierczak et al. 2024). Reported accuracy reflects agreement with the reference standard used in each study and should not be interpreted as equivalence to clinician performance. Applications are not equally supported by evidence: facial asymmetry assessment is included to illustrate that automated analysis does not perform adequately in all diagnostic tasks. Figure created by the authors. Abbreviations: CBCT, cone-beam computed tomography; CI, confidence interval; CNNs, convolutional neural networks; CV, computer vision; DL, deep learning; Dice, Dice similarity coefficient; ICC, intraclass correlation coefficient; IoU, intersection over union; ML, machine learning; OR, odds ratio

Among orthodontic AI applications, diagnostic imaging has accumulated some of the most extensive technical-validation evidence (Fig. 4). By combining DL and CV, AI systems can automate the analysis of orthodontic images and digital datasets, including cephalograms, CBCT scans, intraoral scans, and digital models, although the extent to which such automation improves clinical efficiency, diagnostic decision-making, or patient outcomes depends on the specific application and level of clinical validation (Liu et al. 2023, Bor et al. 2024, Kazimierczak et al. 2024, Monill-Gonzalez et al. 2021).

Automated cephalometric landmark detection is among the earliest and most extensively investigated AI applications in orthodontics, with CNN-based models evaluated in numerous validation studies (Bor et al. 2024, Kazimierczak et al. 2024, Schwendicke et al. 2021). In a systematic review and meta-analysis of 19 studies published between 2017 and 2020, all employing CNNs and predominantly evaluated on two-dimensional (2D) lateral cephalograms, Schwendicke et al. (Schwendicke et al. 2021) reported that 79.9% (95% CI 77.0%-82.4%) of landmarks were detected within the commonly applied 2-mm clinical acceptability threshold, with pooled prediction error centring on that threshold (mean difference − 0.581 mm; 95% CI − 1.264 to 0.102). However, risk of bias was rated high in most included studies, applicability concerns were identified regarding data selection and reference-test conduct, and 12 of the 19 studies relied on publicly available benchmark datasets rather than independent clinical cohorts. These findings indicate that approximately one in five landmarks fell outside the clinically accepted threshold, and that reported performance derives largely from benchmark datasets under retrospective conditions rather than from prospective clinical evaluation. Individual validation studies have nonetheless reported strong agreement between AI-assisted and conventional cephalometric analyses for selected measurements (Bor et al. 2024, Kazimierczak et al. 2024).

Beyond 2D cephalometry, AI has become an integral component of 3D image analysis. In an external validation study of 190 CBCT scans across five CBCT systems, automated anterior tooth segmentation using cloud-based commercial software was rated adequate without manual refinement in 90.1% of 879 tooth segmentation maps, with Dice similarity coefficients (DSC) of 0.96–0.97, Intersection over Union (IoU) of 0.92–0.95, recall of 0.94–0.95, precision and accuracy above 0.97, median absolute distance (MAD) below 0.07 mm, and root mean squared error (RMSE) below 0.10 mm, and was substantially faster than manual segmentation (Adiverci et al. 2026). Notably, the presence of orthodontic brackets was independently associated with an increased need for manual refinement (OR = 3.74, *p* < 0.05), as were endodontic treatment (OR = 4.43, *p* < 0.05) and adjacent high-density artifacts (OR = 7.88, *p* < 0.05), indicating that performance in orthodontic populations may differ from that in general dental cohorts.

AI has similarly been applied to intraoral scans and digital models. A fully automated deep-learning model for tooth segmentation and FDI labelling, developed on 600 full- and partial-arch intraoral scans and evaluated by five-fold cross-validation, achieved tooth Dice coefficients of 0.982 and 0.986 for full- and partial-arch scans respectively, with comparable performance on a public benchmark dataset; errors were significantly associated with residual roots, residual crowns, missing teeth, and partially erupted teeth, indicating high technical performance while also identifying clinical conditions in which segmentation errors may occur (Cao et al. 2025) (Fig. 4). AI has also been applied to digital dental models for automated tooth segmentation and classification of orthodontic conditions, with potential applications in standardized digital assessment (Monill-Gonzalez et al. 2021). Furthermore, AI-based approaches have been investigated for CBCT image analysis and interpretation, with potential to improve the efficiency and consistency of radiographic assessment (Mohammad-Rahimi et al. 2021, Schulze et al. 2024). Deep-learning approaches have also been investigated for classification and analysis of routine orthodontic clinical photographs, extending image-based AI applications beyond radiographic and three-dimensional datasets (Ryu et al. 2022).

AI has also been applied to skeletal maturity and growth assessment. Machine-learning regression models predicting craniofacial growth from serial lateral cephalograms achieved prediction accuracies of 97.87% for linear and 94.45% for angular skeletal parameters at age 13, although the cohort comprised 59 children from a single population (Kim et al. 2023), and comparable approaches have been applied to cervical vertebral maturation staging (Kök et al. 2019). Performance is not uniform across diagnostic tasks. In a comparison of an AI-driven web platform with manual 3D measurement in 95 craniofacial CT examinations, erroneous automated tracings occurred in 16.8% of cases and agreement with manual asymmetry measurement was very low (ICC < 0.3), with no agreement for the asymmetry rate index, leading the authors to conclude that the platform could not be used to assess facial asymmetry (Kazimierczak et al. 2024). Manual measurement repeatability in the same study was excellent (ICC > 0.947), indicating that the discrepancy reflected automated tracing error rather than measurement unreliability.

##### Current evidence and clinical translation

Diagnostic imaging represents the most extensively technically validated area of orthodontic AI. External validation is available for selected applications, particularly CBCT segmentation, but prospective clinical evaluation remains limited. Consequently, although these applications represent the most advanced evidence base identified in this review, they do not yet meet the predefined criteria for higher clinical maturity, which additionally require prospective clinical evaluation. Commercial availability was considered an implementation characteristic rather than evidence of clinical effectiveness or superiority. Accordingly, although diagnostic imaging applications represented the most advanced evidence base identified in this review, no application met the predefined criteria for higher clinical maturity because prospective clinical effectiveness remained insufficiently established.

### Emerging clinical applications

#### AI-assisted treatment planning and virtual treatment simulation

Beyond diagnosis, AI has been investigated for orthodontic treatment-planning tasks using clinical records, radiographs, 3D imaging, and historical treatment data. Current investigated applications of AI in orthodontics include automated treatment planning, virtual tooth-movement simulation, extraction decision support, biomechanical analysis, aligner staging, and orthognathic surgery planning (Gracea et al. 2025, Retrouvey and Conley 2023).

ML models trained on clinical and cephalometric datasets can identify patterns associated with orthodontic treatment and generate predictions relevant to treatment planning or anticipated outcomes based on skeletal morphology, dental relationships, and other patient-specific characteristics (Mohammad-Rahimi et al. 2021, Cho et al. 2024). AI-assisted planning systems have been investigated for various aspects of orthodontic treatment planning, including biomechanical considerations, bracket positioning, wire sequencing, and extraction versus non-extraction decisions (Bichu et al. 2021, Retrouvey and Conley 2023). Extraction decision-making is the most extensively evaluated of these tasks. A systematic review and meta-analysis reported pooled sensitivity of 70% (95% CI 61–78) and pooled specificity of 90% (95% CI 87–92) for AI models predicting the need for orthodontic extraction, with CNNs among the better-performing architectures (Ziaei et al. 2025). Heterogeneity was very high for both estimates (I² = 96.7% and 93.7%, respectively), reflecting substantial variation in model architecture, input data, and reference standards, and limiting the confidence that can be placed in the pooled figures; an earlier meta-analysis reached broadly similar conclusions (Evangelista et al. 2022). Reported accuracy in this domain therefore derives from retrospective model development rather than from evaluation of whether AI-assisted recommendations alter clinical extraction decisions. In addition, AI-driven virtual setups and treatment simulations can visualize or predict aspects of treatment progression and anticipated esthetic changes, and may therefore provide visual decision-support or communication tools; whether they improve informed consent, treatment decisions, or clinical outcomes requires direct evaluation (Dipalma et al. 2023, Cho et al. 2024, Thorat et al. 2024). The availability of increasingly large digital datasets has further enabled personalized treatment planning. By integrating patient-specific clinical and cephalometric characteristics, AI-based systems can support individualized orthodontic treatment and management decisions, although evidence that this translates into improved workflow efficiency, consistency, or treatment outcomes remains limited (Strunga et al. 2023, Kazimierczak et al. 2024).

##### **Current evidence and clinical translation**

AI-assisted treatment planning is evolving rapidly, and several commercial platforms already incorporate automated planning tools. A systematic review of 19 studies evaluating AI-based and knowledge-based systems for orthodontic diagnosis and treatment planning reported accuracies frequently exceeding 80%, and in some studies exceeding 90%, when model outputs were compared with expert decisions (Baxmann et al. 2026). These figures require careful interpretation, because expert opinion served as the reference standard in most included studies; reported accuracy therefore reflects agreement with clinician judgement rather than demonstrated correctness of the treatment decision. Risk of bias, assessed using ROBINS-I, was predominantly moderate to serious, attributed to retrospective designs, single-centre samples, limited external validation, and reliance on expert opinion as the reference standard. The review also identified a trade-off between approaches: rule-based, fuzzy, and Bayesian systems offered greater transparency of reasoning, whereas data-driven models generally achieved higher discriminative performance. Current evidence in this area therefore remains derived predominantly from retrospective analyses, proof-of-concept studies, and technical validation investigations. Although these systems demonstrate promising computational performance, robust prospective clinical studies demonstrating improvements in treatment quality, treatment duration, or patient-centered outcomes remain scarce. Consequently, AI should currently be regarded as a clinical decision-support tool rather than an autonomous treatment-planning system, and reported agreement with expert decisions should not be interpreted as demonstrated superiority over conventional treatment planning.

#### AI-Assisted treatment monitoring

AI is being increasingly investigated for orthodontic treatment monitoring by enabling automated or remote assessment of treatment progress. Unlike conventional visit-based follow-up, AI-enabled remote monitoring can analyze patient-generated data, including intraoral photographs and digital records, to assess treatment progress and potentially support assessment of appliance use and adherence between clinical visits (Strunga et al. 2023). Sensor-enabled orthodontic appliances have been investigated for direct measurement of intraoral forces. A flexible 3D sensor system has been developed and validated under laboratory conditions for monitoring orthodontic force magnitude and direction (Lee et al. 2022), and IoT-based platforms have been proposed to enable wireless, near-real-time transmission of intraoral force data (Merenda et al. 2020). These systems remain at the prototype stage, and their integration with machine-learning analysis of treatment progression has not yet been demonstrated in routine clinical practice (Merenda et al. 2020, Lee et al. 2022).

##### Current evidence and clinical translation

AI-assisted monitoring is an expanding area of digital orthodontics, with several commercially available remote monitoring platforms, although the extent of routine clinical uptake has not been documented. However, most available evidence is derived from retrospective studies, feasibility investigations, or technical validation studies. Robust prospective clinical trials demonstrating improvements in treatment efficiency, patient adherence, or long-term clinical outcomes remain limited, and the predictive performance of many monitoring algorithms requires further external validation. The monitoring workflow should therefore be interpreted as a conceptual framework, in which several proposed capabilities and downstream benefits remain to be demonstrated in prospective clinical studies.

#### AI for patient communication and education

AI is also being investigated for patient communication and education in orthodontics by providing interactive and potentially personalized information throughout treatment. AI-powered chatbots can answer common patient questions, provide oral hygiene and treatment-related guidance, and provide information between clinical visits (Santonocito et al. 2025, Thorat et al. 2024).

In a preliminary randomized controlled trial, Santonocito et al. (Santonocito et al. 2025) allocated 100 orthodontic patients to receive either printed information leaflets or access to an AI-based chatbot developed from specialty society guidelines. Both groups showed a statistically significant deterioration in plaque index and modified gingival index over five weeks of treatment (*P* < 0.001); however, the increase in modified gingival index was significantly smaller in the chatbot group than in the control group (*P* < 0.001). Within the chatbot group, patients treated with fixed appliances showed a greater increase in gingival index than those treated with clear aligners (*P* < 0.001). These findings suggest that chatbot-delivered instruction may attenuate, rather than prevent, the decline in gingival health commonly observed during orthodontic treatment. The short five-week follow-up, the single-center design, and the absence of a reported between-group difference in plaque index limit the generalizability of these results. Findings across comparative evaluations are inconsistent. Salmanpour et al. (Salmanpour et al. 2025) reported favorable performance for ChatGPT-4.0 on general orthodontic questions, whereas Dursun and Bilici Geçer (Dursun and Bilici Geçer 2024), assessing clear aligner–related questions, found significantly higher modified DISCERN and global quality scores for Microsoft Copilot. These discrepancies may reflect differences in question sets, evaluation instruments, model versions, and assessment dates. Interpretation of these comparisons is complicated by the fact that Microsoft Copilot was, at the time of evaluation, built on the same underlying GPT-4 model as ChatGPT-4 but augmented with a retrieval and source-citation layer. Observed differences may therefore reflect the retrieval architecture and interface design rather than differences in the underlying language model, illustrating why studies evaluating conversational AI should report the specific model, interface, and configuration assessed.

Beyond text-based communication, AI-enabled visualizations, including virtual treatment simulations, treatment animations, and 3D educational models such as OralViewer, have been investigated as tools for visualizing dental and orthodontic procedures and anticipated treatment outcomes; evidence that these tools improve patient understanding, informed consent, or treatment outcomes remains limited (Albalawi and Abalkhail 2022, Liang et al. 2021).

AI has further been integrated with smartphone-based and remote-monitoring approaches through applications that enable remote monitoring of orthodontic treatment progress, adherence, aligner or appliance status, and oral hygiene. Sensor-enabled orthodontic appliances have also been investigated for monitoring treatment-related forces and appliance performance (Strunga et al. 2023, Lee et al. 2022). By monitoring treatment progress against predefined treatment goals, these systems can provide individualized feedback that may support patient engagement and adherence; however, these downstream benefits require prospective clinical confirmation throughout orthodontic care (Gracea et al. 2025, Strunga et al. 2023).

Generative AI has also been evaluated in orthodontic education. A systematic review of 17 studies reported evidence suggesting improvements in knowledge retention and clinical decision-making through adaptive learning pathways and real-time feedback, with risk of bias assessed using the Cochrane Risk of Bias Tool and the Newcastle-Ottawa scale and evidence quality appraised using GRADE (Baxmann et al. 2025). Because the search underpinning that review closed in December 2023, its findings predate the current generation of LLMs, and its conclusions should be interpreted in light of the version dependency discussed in Sect.  3.5.

##### Current evidence and clinical translation

The orthodontic chatbot evaluations identified in this review were published before or were not designed according to the subsequently available CHART reporting recommendations, and reporting of model identifiers, prompt strategies, query procedures, and evaluation dates was often incomplete (The CHART Collaborative 2025). AI-assisted communication and education may facilitate information delivery and patient interaction. However, evidence regarding their clinical effectiveness remains limited, with much of the current orthodontic literature consisting of cross-sectional and comparative evaluations rather than clinical trials. The rapid emergence of generative AI also raises important concerns regarding factual accuracy, hallucinations, patient privacy, and medicolegal responsibility (Yu et al. 2023, Price and Cohen 2019). Performance on information-quality instruments or expert-rated question sets should not be interpreted as evidence that unsupervised LLM-generated patient counseling is safe or clinically effective. Consequently, AI-generated information should complement rather than replace professional communication, clinical expertise, and judgment (Salmanpour et al. 2025, Thorat et al. 2024).

### Experimental applications

#### AI-Driven robotics and automated orthodontic systems

The integration of AI with robotic technologies represents an experimental area in orthodontics. Robotic and automated systems have been investigated for tasks including customized archwire fabrication and appliance positioning; however, automation or robotic execution should not itself be considered evidence that a system uses AI (Adel et al. 2021, Albalawi and Abalkhail 2022, Fawaz et al. 2023, Nordblom et al. 2024).

Digitally controlled robotic wire-bending systems can translate planned arch forms into customized archwires, but the extent to which individual systems incorporate AI rather than conventional rule-based automation should be distinguished explicitly (Adel et al. 2021). Robotic or automated approaches have also been investigated for appliance positioning and other components of digital orthodontic workflows; evidence of improved clinical precision, chairside efficiency, or treatment outcomes remains limited and should not be inferred from technical automation alone (Adel et al. 2021, Kuzyk and Kotelban 2023). In clear aligner therapy, AI has been applied to or investigated for digital tooth segmentation, virtual tooth movement, treatment simulation and staging, and individualized treatment planning (Ruiz et al. 2025, Retrouvey and Conley 2023, Kazimierczak et al. 2024). Reinforcement-learning approaches have been explored more broadly in oral healthcare, but evidence for their direct clinical application to sequential orthodontic treatment optimization remains insufficient (Trella et al. 2023).

##### Current evidence and clinical translation

Robotic and automated orthodontic systems have demonstrated technical feasibility for selected tasks, but the orthodontic evidence base remains sparse and is dominated by proof-of-concept, laboratory, and technical reports. Evidence specifically evaluating the incremental contribution of AI to robotic systems is even more limited, and prospective evidence demonstrating improved clinical outcomes has not been established. High implementation costs, workflow complexity, and regulatory challenges currently limit widespread clinical adoption.

#### Integration with emerging digital technologies

The future of AI-assisted orthodontics may involve integration with complementary digital technologies, including the IoT, augmented reality (AR), virtual reality (VR), cloud computing, and other connected digital platforms. These technologies are not themselves subsets of AI but may provide data-acquisition, visualization, connectivity, or interaction layers for AI-enabled systems. IoT-enabled intraoral sensing technologies may facilitate continuous or near-real-time acquisition and wireless transmission of orthodontic force data, although evidence that such sensing improves orthodontic monitoring or treatment outcomes remains preliminary (Merenda et al. 2020, Mohammad-Rahimi et al. 2021, Lee et al. 2022). AR and VR technologies have been investigated as potential complements to AI-assisted orthodontic workflows by enabling interactive visualization of treatment plans, surgical simulations, and immersive educational environments (Uruthiralingam and Rea 2020, Marya et al. 2025). Although these technologies have been more extensively explored in implant dentistry and orthognathic surgery, they are also being investigated in orthodontic contexts for diagnosis (Joda et al. 2019), treatment planning and simulation, patient education, and clinical training (Marya et al. 2025). Future integration of AI, robotics, AR/VR, and cloud-based computing could theoretically enable greater integration of patient-specific data, treatment simulation, and longitudinal monitoring; however, such integrated adaptive workflows remain largely conceptual in orthodontics (Samaranayake et al. 2025, Nordblom et al. 2024).

##### Current evidence and clinical translation

Most integrated AI ecosystems remain experimental. While preliminary studies demonstrate technical feasibility and educational value, clinical implementation is still limited by high costs, technological complexity, interoperability challenges, and the lack of standardized validation protocols. Their impact on treatment efficiency and patient outcomes has yet to be confirmed in prospective clinical studies. Evidence from adjacent dental or surgical disciplines should not be interpreted as direct evidence of orthodontic clinical effectiveness.

### Future research priorities

The continued evolution of AI in orthodontics will require close collaboration between orthodontists, computational scientists, engineers, and other clinical and behavioral specialists to ensure that AI systems are technically robust, clinically relevant, and responsive to patient needs (Gracea et al. 2025, Nordblom et al. 2024). Future research should prioritize large, diverse, and representative multicenter datasets; rigorous external validation and explainability of AI models; standardized reporting and evaluation frameworks; and prospective clinical studies assessing clinically relevant and patient-centered outcomes (Gracea et al. 2025, Monill-Gonzalez et al. 2021, Nordblom et al. 2024). As AI becomes increasingly integrated into digital orthodontic workflows, clinicians will require greater proficiency in interpreting AI-generated information and using digital technologies, alongside interdisciplinary collaboration, while retaining responsibility for clinical decision-making and patient care (Samaranayake et al. 2025, Gracea et al. 2025, Nordblom et al. 2024).

#### Current evidence and clinical translation

Future progress in AI will depend not only on improvements in technical performance but increasingly on generating robust clinical evidence demonstrating meaningful benefits for treatment quality, efficiency, safety, and patient outcomes. Large multicenter prospective studies, independent external validation, clinically meaningful outcome measures, and transparent reporting standards will therefore be essential before many experimental AI applications can be routinely implemented (Table 3).

**Table 3:** Evidence synthesis and clinical maturity of artificial intelligence (AI) applications in orthodontics

Application	Technical evidence	External validation	Prospective clinical evidence	Demonstrated benefit	Commercial status	Maturity
**Cephalometric landmark detection**	Meta-analysis of 19 CNN-based studies: 79.9% of landmarks (95% CI 77.0–82.4) detected within 2 mm; pooled mean difference − 0.581 mm (95% CI − 1.264 to 0.102).	Partial. Independent comparative evaluations are available; however, 12/19 studies in the meta-analysis used public benchmark datasets rather than independent clinical cohorts.	None identified.	Not demonstrated. No identified evidence of altered treatment decisions, reduced chair time, or patient-reported benefit.	Yes. Multiple commercial platforms.	Emerging. Strong technical evidence with some independent validation, but no prospective clinical effectiveness. Refs: 16,17,33.
**CBCT segmentation**	In 190 CBCT scans, 90.1% of 879 segmentation maps were adequate without manual refinement; Dice 0.96–0.97; IoU 0.92–0.95; median absolute distance < 0.07 mm. Manual refinement was more likely with brackets (OR 3.74), endodontic treatment (OR 4.43), and adjacent high-density artefacts (OR 7.88).	Yes. Multicentre external validation across five CBCT systems; strongest external-validation evidence identified in this review.	None identified.	Limited. Time saving relative to manual segmentation demonstrated; no patient- or health-system outcomes reported.	Yes. Cloud-based commercial software.	Emerging. Strong technical and external-validation evidence, but prospective clinical effectiveness remains unestablished. Refs: 41,43,53.
**Tooth segmentation and labelling of intraoral scans and digital models**	Fully automated segmentation and FDI labelling developed on 600 scans: Dice 0.982 (full arch) and 0.986 (partial arch) under five-fold cross-validation. Errors were associated with residual roots and crowns, missing teeth, and partially erupted teeth.	Limited. Comparable performance reproduced on a public benchmark dataset; no independent clinical cohort or multicentre validation identified.	None identified.	Not demonstrated.	Yes. Integrated into digital diagnostic and treatment-planning software.	Emerging. High technical performance, but insufficient independent clinical validation. Refs: 26,42,47.
**AI-assisted remote treatment monitoring**	Feasibility and platform-level evidence available; no pooled accuracy estimate for commercial platforms identified. Sensor-based intraoral force measurement validated only under laboratory conditions.	None identified for commercial monitoring platforms.	None identified.	Not demonstrated. Reduced visit frequency, improved treatment adherence, and optimized treatment timing remain proposed rather than demonstrated benefits.	Yes. Several commercial remote-monitoring platforms.	Emerging. Feasibility evidence exists, but external validation and prospective clinically meaningful effectiveness remain limited. Refs: 11,52,55.
**AI-assisted treatment planning and virtual setups**	Variable and task-dependent. For extraction decision-making, pooled sensitivity 70% (95% CI 61–78) and specificity 90% (95% CI 87–92), with very high heterogeneity (I²=96.7% and 93.7%). Other tasks rely mainly on retrospective or proof-of-concept studies.	Limited. A systematic review of 19 studies identified limited external validation, single-centre sampling, and reliance on expert opinion as recurring weaknesses.	None identified.	Not demonstrated. No identified evidence evaluating treatment quality, treatment duration, patient-centred outcomes, or whether AI recommendations alter clinical decisions.	Partial. Planning and virtual-setup modules are embedded in aligner and treatment-planning platforms.	Emerging. Technical evidence is heterogeneous and prospective clinical effectiveness remains unestablished. Refs: 7,15,27,49,50,54.
**Generative AI for patient education and communication**	Evaluated mainly using information-quality instruments and expert ratings rather than conventional accuracy metrics. Comparative model rankings are inconsistent and dependent on question sets, instruments, model versions, and assessment dates.	Limited. Direct comparison and replication across proprietary versions is constrained by model updating, incomplete versioning, and configuration dependence.	Limited. One preliminary single-centre RCT (*n* = 100; five-week follow-up) compared a guideline-derived chatbot with printed information leaflets.	Partial and modest. Increase in modified gingival index was smaller with the chatbot (*P* < 0.001); plaque index deteriorated in both groups without a reported between-group difference.	Partial. General-purpose conversational tools used off-label; no validated orthodontic-specific patient-education product identified.	Emerging. Only application with prospective randomized evidence identified, but evidence is preliminary, short-term, single-centre, and based on a surrogate periodontal outcome. Refs: 34,38,39.
**Treatment outcome prediction**	Mean prediction error 2.12 mm; AI outperformed conventional methods for 5/44 landmarks (11.4%).	None identified.	None identified.	Not demonstrated.	None identified.	Experimental. Evidence derives from a small number of comparative and modelling studies using single-centre retrospective datasets, without externally validated prediction models. Refs: 48,58.
**Generative AI for clinical decision support and documentation**	Orthodontic-specific performance evidence is insufficient. Available evaluations are extrapolated mainly from general healthcare informatics; performance is sensitive to model version, prompting, conversational context, and sampling variability.	None identified in orthodontics. Reproducibility and temporal generalizability are limited by proprietary model updates and configuration dependence.	None identified.	Not demonstrated. No identified evidence of effects on documentation time, diagnostic accuracy, or clinical decisions in orthodontics.	None identified for validated orthodontic-specific applications.	Experimental. No orthodontic primary study of clinical decision support or documentation was identified. Ref: 6.
**Robotics and automated orthodontic systems**	Laboratory and proof-of-concept work addresses archwire bending, bracket positioning, and aligner manufacturing; quantitative performance evidence is sparse.	None identified.	None identified.	Not demonstrated.	Limited. Wire-bending systems available in specialized settings.	Experimental. Evidence remains largely laboratory or prototype based, with sparse evidence demonstrating an AI component or incremental clinical benefit. Ref: 1.
**AI integration with emerging digital technologies (AR**,** VR**,** and IoT)**	Predominantly feasibility and prototype evidence. IoT platforms have been proposed for near-real-time intraoral force transmission; flexible sensor systems have laboratory validation. AR/VR evidence relates mainly to visualization, simulation, and education.	None identified.	None identified in orthodontics.	Not demonstrated. Educational value reported in adjacent fields, but no orthodontic patient-, clinician-, or health-system outcomes identified.	None identified for orthodontics-specific integrated systems.	Experimental. Evidence is largely prototype-based or extrapolated from adjacent dental/medical fields; clinical AI integration has not been demonstrated. Refs: 10,11,55,60,61.

Note. Technical evidence refers to task-specific performance or feasibility. External validation refers to evaluation beyond the development setting or dataset. Prospective clinical evidence refers to prospective evaluation under clinical conditions. Demonstrated clinical/patient benefit refers to evidence of effects on clinical decisions, workflow, patient outcomes, or health-system outcomes. Commercial implementation is reported as an implementation characteristic only and was not considered evidence of clinical effectiveness, superiority, or maturity. “None identified” indicates that no eligible evidence was identified in this review. No application satisfied the criteria for an established clinical application. Abbreviations: AI, artificial intelligence; AR, augmented reality; CBCT, cone-beam computed tomography; CI, confidence interval; CNN, convolutional neural network; Dice, Dice similarity coefficient; FDI, Fédération Dentaire Internationale; IoT, Internet of Things; IoU, intersection over union; OR, odds ratio; RCT, randomized controlled trial; VR, virtual reality

## Challenges and future perspectives

**Fig. 5:**
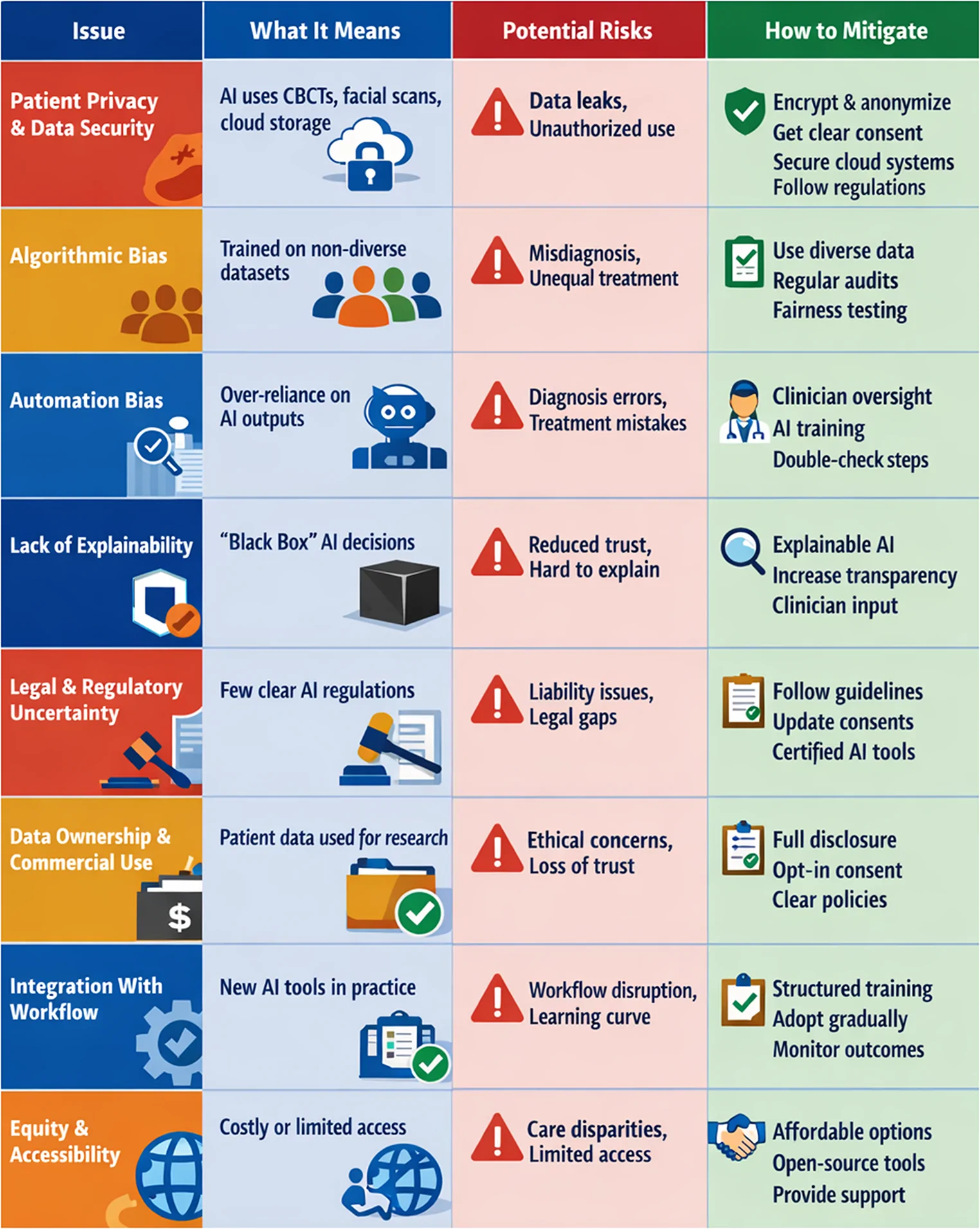
Ethical, legal, clinical, and practical considerations for artificial intelligence implementation in orthodontics. The figure summarizes major considerations relating to data governance, bias and generalizability, transparency, clinician oversight, regulation, and responsible clinical implementation. Figure created by the authors

Despite its considerable potential, the successful integration of AI into orthodontics depends not only on technical performance but also on addressing important ethical, legal, clinical, and methodological challenges (Fig. 5). Although AI has demonstrated high technical performance in selected diagnostic tasks, widespread clinical adoption remains constrained by limited prospective and real-world clinical evidence, concerns regarding data quality and generalizability, regulatory uncertainty, and the need for greater transparency, explainability, and rigorous evaluation of AI-assisted decision-making (Albalawi and Abalkhail 2022, Gracea et al. 2025, Nordblom et al. 2024). Importantly, technical performance alone does not establish external generalizability, prospective clinical effectiveness, or meaningful benefit to patients, clinicians, or health systems.

### Ethical, legal, and clinical challenges

AI systems rely on large volumes of sensitive patient data, including radiographs, 3D dental models, facial photographs, and electronic health records. Consequently, data privacy, cybersecurity, informed consent, and secondary use of patient data remain major concerns. Even anonymized datasets may be vulnerable to re-identification through cross-referencing, particularly when facial imaging is involved, emphasizing the need for robust data governance and transparent consent procedures (Price and Cohen 2019, Nordblom et al. 2024).

The reliability of AI systems also depends heavily on the quality, representativeness, and diversity of their training datasets. Models developed from geographically or ethnically homogeneous populations may perform less accurately in underrepresented patient groups, potentially introducing algorithmic bias and diagnostic disparities (Obermeyer et al. 2019). Independent external validation across diverse populations, institutions, and acquisition environments, transparent reporting, and ongoing monitoring of algorithm performance, generalizability, and potential bias should be established as prerequisites for responsible clinical implementation (Obermeyer et al. 2019, Nordblom et al. 2024). Performance should also be monitored after deployment because changes in patient populations, clinical workflows, imaging systems, or model behavior may affect real-world performance over time.

Another important challenge is automation bias, whereby clinicians may over-rely on AI-generated recommendations without critically evaluating the outputs (Goddard et al. 2012). AI should therefore be regarded as a clinical decision-support tool rather than a replacement for professional judgment (Nordblom et al. 2024, Salmanpour et al. 2025). Improving AI literacy through undergraduate education and continuing professional development will be essential to enable clinicians to critically interpret and appropriately verify AI-assisted outputs (Samaranayake et al. 2025, Nordblom et al. 2024, Schwendicke et al. 2023). Explainability may facilitate interpretation of model outputs but should not itself be considered evidence of model validity, safety, or clinical effectiveness.

The regulatory landscape for AI in healthcare is also evolving. Although regulatory agencies such as the U.S. Food and Drug Administration (FDA) have developed an evolving regulatory framework for AI/ML-enabled medical devices, dedicated and comprehensive regulatory frameworks for dental and orthodontic AI remain comparatively limited. Clear standards addressing algorithm validation, software modifications and updates, transparency, accountability, and medicolegal responsibility will be important to ensure safe and responsible implementation in clinical practice (Nordblom et al. 2024, Food , Cestonaro et al. 2023). Commercial availability or regulatory authorization should likewise be distinguished from evidence that an AI system improves clinical outcomes or is superior to conventional care.

### Synthesis of evidence across applications

Across the applications reviewed, three patterns emerge. First, selected image-analysis applications—particularly cephalometric landmark detection and cone-beam computed tomography (CBCT) segmentation—have accumulated comparatively strong technical-validation evidence, although the extent of independent external validation varies substantially between tasks. Technical performance is also high for tooth segmentation from intraoral scans, but independent clinical validation remains more limited. Across these applications, prospective evidence demonstrating improved treatment decisions or patient outcomes was not identified. Second, heterogeneity is particularly evident in evaluations of conversational AI, where comparative studies have reached opposing conclusions regarding model performance, differences that may be attributable to question sets, evaluation instruments, model versions, assessment dates, prompting procedures, and system configurations, rather than reflecting stable differences between underlying models. Third, the principal translational evidence gaps are consistent across application domains: no identified study links AI-assisted diagnosis to a change in treatment decisions; orthodontic-specific NLP evidence remains minimal, with most applications extrapolated from general healthcare informatics; and sensor-based and robotic systems remain predominantly confined to prototype and laboratory settings. Overall, the evidence decreases progressively when moving from technical performance to independent external validation, prospective clinical effectiveness, and demonstrated patient- or system-level benefit. Commercial implementation is more advanced for some applications, but availability alone does not resolve these evidence gaps. Across application domains, a consistent finding was that technical validation has advanced further than evidence of real-world clinical effectiveness. Thus, the principal area of convergence in the literature is not that AI has established clinical benefit, but that selected applications can achieve strong task-specific technical performance; whether this performance generalizes across settings or improves clinical decisions, workflow, or patient outcomes remains insufficiently established.

### Future perspectives

Future advances in AI-assisted orthodontics will depend not only on continued technical development but increasingly on progression from technical performance to independent external validation, prospective clinical effectiveness, and demonstrated clinically meaningful benefit. Most current AI applications have been evaluated using retrospective datasets or technical validation studies, whereas prospective multicenter clinical studies demonstrating improvements in treatment quality, efficiency, patient adherence, safety, clinician decision-making, workflow performance, patient-reported outcomes, or long-term outcomes remain scarce (Gracea et al. 2025, Nordblom et al. 2024).

Further progress will require the development of large, diverse, and well-annotated datasets, preferably through multicenter collaboration, to improve model generalizability across different patient populations, imaging systems, and clinical environments. In parallel, greater emphasis should be placed on explainable AI, transparent reporting standards, independent external validation, prospective evaluation, post-deployment monitoring, and effective integration with existing digital orthodontic workflows. Emerging computational and digital technologies may further expand the role of AI in orthodontics by supporting the investigation of more personalized, adaptive, and data-informed approaches to treatment planning and monitoring (Samaranayake et al. 2025, Monill-Gonzalez et al. 2021, Nordblom et al. 2024).

Ultimately, the future of AI in orthodontics will depend on interdisciplinary collaboration among orthodontists, engineers, computer scientists, and regulatory authorities to ensure that technological innovation translates into clinically meaningful improvements in patient care rather than simply higher computational performance.

**Key future research priorities include**:

- conducting prospective multicenter clinical studies evaluating clinically meaningful patient-, clinician-, workflow-, and health-system outcomes;
- improving independent external validation using diverse and representative datasets and clinical environments;
- developing explainable and transparent AI models while evaluating whether explainability meaningfully supports safe clinical interpretation and decision-making;
- adopting appropriate established reporting and evaluation frameworks, including TRIPOD + AI, STARD-AI, DECIDE-AI, FUTURE-AI, TRIPOD-LLM, and CHART, in orthodontic AI research, according to the study design and AI application for which each framework was developed;
- prospectively evaluating generative AI, robotics, and multimodal digital workflows before their integration into routine orthodontic practice; and.
- establishing mechanisms for post-deployment evaluation, including monitoring of real-world performance, safety, bias, and changes associated with model or software updates.

### Limitations

Several limitations should be considered when interpreting this review. First, the review was conducted as a narrative synthesis rather than a systematic review. Publications were selected according to the predefined eligibility and selection procedures described in Sect.  2.3, and selection bias cannot be excluded.

Second, no formal risk-of-bias or quality-appraisal instrument was applied. Applications were classified by clinical maturity according to the predefined criteria described in Sect.  2.5, but individual studies were not formally scored for adherence to individual items within contemporary reporting frameworks. Conclusions regarding evidence strength should therefore be interpreted as a structured qualitative appraisal rather than a formal quality assessment. The clinical-maturity framework used in this review was developed as a structured narrative appraisal and should not be interpreted as a validated quantitative scoring instrument.

Third, the search was restricted to PubMed, ScienceDirect, and the Cochrane Library. Scopus, Embase, and Web of Science were not searched, and relevant engineering and computational literature published in non-indexed conference proceedings, technical reports, or manufacturer documentation may therefore have been missed. Publications in languages other than English were not considered. In addition, ScienceDirect is a publisher platform rather than a comprehensive bibliographic database, which may further limit coverage of engineering and computational literature.

Fourth, outcomes reported across the included literature were highly heterogeneous, encompassing landmark detection error, segmentation overlap coefficients, information-quality scores, and clinical indices. This heterogeneity precluded quantitative synthesis and limits direct comparison between applications.

Finally, evidence concerning generative AI and LLMs is evolving rapidly. Because proprietary models may undergo updates or configuration changes that are incompletely documented or not publicly versioned, findings reported for a given model release may not apply to its successor, and conclusions drawn in this domain are likely to date more quickly than those concerning image-analysis applications. Differences in prompts, interfaces, retrieval functions, evaluation dates, and sampling behavior may further limit reproducibility and direct comparison between LLM studies.

## Conclusion

AI is increasingly being investigated across orthodontic diagnosis, treatment planning, monitoring, documentation, and patient communication, but the maturity of evidence varies substantially between applications. Among current applications, automated cephalometric landmark detection and CBCT segmentation have accumulated comparatively strong technical-validation evidence, with external validation available for selected tasks. Tooth segmentation from intraoral scans and digital models has also demonstrated high technical performance, although independent clinical validation remains more limited. Commercial implementation is available for several applications but should not be interpreted as evidence of clinical effectiveness or superiority. Other emerging or experimental applications, including AI-assisted treatment planning, remote monitoring, generative AI, and robotic systems, remain supported predominantly by retrospective, technical-validation, feasibility, or early clinical evidence, with substantial variation between application domains.

However, the current evidence base remains dominated by retrospective studies, technical validation investigations, and proof-of-concept reports. While many AI systems demonstrate high technical performance for selected tasks, robust prospective clinical evidence demonstrating improvements in treatment quality, efficiency, patient adherence, clinical decision-making, safety, patient-reported outcomes, or long-term outcomes remains limited. Across the evidence reviewed, technical validation has generally progressed further than external validation and prospective clinical effectiveness, while demonstrated patient- or health-system benefit remains largely unestablished. Consequently, AI should currently be regarded primarily as a clinician-supervised decision-support technology that complements, rather than replaces, orthodontic expertise.

Future progress will depend on the development of large, diverse, and externally validated datasets, explainable AI models, transparent and study-appropriate reporting, and well-designed multicenter prospective clinical studies evaluating clinically meaningful outcomes. Equally important are robust ethical and regulatory frameworks that ensure transparency, patient privacy, accountability, safety, and responsible implementation. Progression toward routine clinical implementation should therefore be guided by evidence that extends beyond algorithmic accuracy to demonstrate generalizability, prospective clinical effectiveness, and meaningful benefit, while preserving clinician responsibility for patient management.

## Data Availability

No datasets were generated or analysed during the current study.

## Abbreviations

2D Two: Dimensional
3D Three: Dimensional
AI: Artificial intelligence
ANNs: Artificial neural networks
AR: Augmented reality
CBCT: Cone-beam computed tomography
CNNs: Convolutional neural networks
CV: Computer Vision
DL: Deep Learning
FDA: U.S. food and drug administration
GANs: Generative adversarial networks
IoT: Internet of things
LLMs: Large language models
ML: Machine learning
NLP: Natural language processing
VR: Virtual reality
ICC: Intraclass correlation coefficient
CI: Confidence interval
DSC: Dice similarity coefficient
IoU: Intersection over union
MAD: Median absolute distance
RMSE: Root mean squared error
FDI: Fédération dentaire internationale
OR: Odds ratio
RCT: Randomised controlled trial
TRIPOD+AI: Transparent reporting of a multivariable prediction model forindividual prognosis or diagnosis + artificial Intelligence
STARD-AI: Standards for reporting of diagnostic accuracy studies -artificial intelligence
DECIDE-AI: Developmental and exploratory clinical investigations of decision support systems driven by artificial intelligence
FUTURE-AI: Fairness, universality, traceability, usability, robustness, explainability
TRIPOD-LLM: Transparent reporting of a multivariable prediction model forindividual prognosis or diagnosis - large language models
DISCERN: A validated instrument name for judging the quality of consumerhealth information (developed at Oxford)
CHART: Chatbot assessment reporting tool

## Declarations

### Generative AI and AI-assisted technologies in the writing process

During the preparation of this work, the authors used ChatGPT and Gemini to improve the readability and language of the manuscript text. No generative AI tools were used in the creation of any figure; all figures were produced by the authors using Canva. After using these tools, the authors reviewed and edited the content as needed and take full responsibility for the content of the publication.
